# Evaluation of the Nutritional Knowledge in Relation to Secondary Prevention Among Doctors and Nurses in the Northern of Morocco: A Cross-Sectional Study

**DOI:** 10.7759/cureus.68689

**Published:** 2024-09-05

**Authors:** Zohra Ben Allal, Nisrin El Mlili, Adil Najdi

**Affiliations:** 1 Laboratory of Epidemiology and Public Health, Faculty of Medicine and Pharmacy Tangier, Abdelmalek Essaâdi University, Tangier, MAR; 2 Public Health and Social Sciences, Higher Institute of Nursing and Health Techniques of Tetouan, Tetouan, MAR; 3 Health Sciences, Higher Institute of Nursing and Health Techniques of Tetouan, Tetouan, MAR; 4 Laboratory of Biology and Health, Faculty of Sciences of Tetouan, Abdelmalek Essaâdi University, Tetouan, MAR; 5 Public Health and Social Sciences, Mohammed VI University Hospital Center of Tangier, Tangier, MAR

**Keywords:** doctor, non-communicable diseases, nurse, nutritional knowledge, secondary prevention

## Abstract

Background: Morocco is a country that has been experiencing an epidemiological and health transition characterized by a change in lifestyle and an increase in mortality for non-communicable diseases (NCDs). The objective of this study is to evaluate the nutritional knowledge in relation to secondary prevention of doctors and nurses working in the hospital and primary healthcare centers in Morocco. Specific knowledge about nutrition for diabetes, for high blood pressure and high cholesterol, for functional colopathy, for iron deficiency anemia, for vitamin B12 deficiency, for overweight and for hyperuricemia or gout attack were evaluated.

Materials and methods: Cross-sectional exploratory study carried out between June and December 2022, using a self-administered questionnaire developed on the basis of similar studies and the several standardized nutrition guides. A random cluster survey included 238 nurses and 131 doctors working in health centers and 234 nurses and 54 doctors working in hospitals. Statistical analyses were carried out using SPSS version 21.0 software, the Kolmogorov-Smirnov test, the Mann Whitney test, and Spearmen's correlation. Multiple linear regression analysis was used to explore the most significant sociodemographic variables.

Results: Among the seven nutritional content areas assessed in this study, we found that among doctors, the nutrition axis for high blood pressure and high cholesterol had the median correct response score of (0.57 interquartile range (IQR) (0.28, 0.71)). The same for nurse, but with the lower scores ((0.28 IQR (0, 0.43)) , (0.28 IQR (0.07, 0.42)) respectively).

Our results do not reveal any statistically significant association of the median of total score of answers of both health professionals (nurses or of doctors) with their gender (p=0.383). However, they were significantly associated with basic training (p<0.001), continuous training (p=0.002), receiving information on nutrition (p=0.018), and their age (p=0.016).

Conclusions and implications: In conclusion, this study highlighted the major gaps in the nutritional knowledge of doctors and nurses regarding secondary prevention of NCDs. However, they should undergo continuing nutrition education programs to ensure safe and sound nutritional advice not only to patients but also to the public. Furthermore, policymakers should devote systematic efforts to nutrition education during basic training in medical and nursing schools, which will provide doctors and nurses with adequate training.

## Introduction

According to the World Health Organization (WHO), non-communicable diseases (NCDs) are responsible for 74% of total deaths each year [1]. The most common conditions, including cardiovascular disease, type 2 diabetes, hypertension and many cancers, are linked, at least in part, to poor diet [2]. In most countries, healthy diets rich in fiber and vitamins have been replaced by unhealthy processed foods high in sugars, fats, animal foods and refined carbohydrates [3].

A poor diet leads to a deficiency of vitamins and minerals, particularly vitamins B12 and iron, which regulate the activation of the complement system and the release of pro-inflammatory cytokines, and leads to low-grade inflammation [4]. Internationally, there is growing concern that poor diet increases potential risk and leads to chronic diseases [3]. The success of treatments and interventions used to combat these diseases depends on improving diet and nutritional status [5].

Nutritional care refers to practices that may include nutritional assessment and nutritional counseling as part of routine consultations [6]. Nutritional care provided by doctors and nurses has been shown to improve patients' eating behaviors [6].

In Morocco, the nutritional transition is leading to a change in eating habits. Research carried out in this country has shown that only 28.2% of the urban population opts for a Mediterranean diet, in addition to excessive consumption of carbohydrates (43 %), and overconsumption of lipids (18%) [7,8]. At the same time, NCDs constitute the main cause of mortality in Morocco, it is estimated at 84% according to the WHO in 2022 [1]. According to the Moroccan directorate of epidemiology and disease control, the prevalence of hypertension is 29.3%, diabetes 10.6%, 10.5% had a rate of high total cholesterol and 33% are overweight and 20% are obese [9].

On the other hand, the situation in the world with regard to the development of nutritional education is far from being up to par. In the United States, only 25% of medical schools include any element of nutrition education in their curriculum [10]. They do not provide adequate nutrition education, nor do they produce graduates with the nutrition skills required in medical practice. Many healthcare providers in this country are not adequately trained to meet lifestyle recommendations that include nutrition behaviors.

The need for nutritional education has, therefore, become crucial. Frontline healthcare professionals, such as doctors, nurses, midwives, and others, can positively impact patient care by synchronizing nutrition education across specialty areas [11]. For this reason, their knowledge of nutrition must be adapted to face numerous obstacles in providing optimal care to patients with chronic diseases. One of the main obstacles lies in the complex interaction of physical, psychological and social factors that impact nutritional status. Therefore, a comprehensive and patient-centered approach to healthcare is necessary.

After consulting the training courses of the faculties of medicine and nursing in Morocco, we noticed that there are no well-developed nutrition modules, apart from a few basic notions linked to certain pathologies and a few educational behaviors to follow.

We aim through this study to evaluate the nutritional knowledge of doctors and nurses in the field of nutrition for hypertension and hypercholesterolemia, diabetes, overweight, functional colopathy, iron-deficiency anemia, vitamin B12 deficiency, anemia, and gout attacks or excess uric acid. The results obtained would make it possible to highlight the shortcomings of basic educational and continuing education programs and to guide educational policies for better preparation of health professionals capable of providing preventive and therapeutic nutritional education to patients for NCDs in Morocco.

## Materials and methods

Study design

This is a cross-sectional exploratory study, which was carried out between June and December 2022.

Sample size calculation and sampling technique

The sample size was calculated based on equations developed by Cochrane as n=Z 2 *PQ/e, then adjusted for the completeness factor $&radic; N-n /N-1$ [12]. 238 nurses and 131 doctors working in primary healthcare centers and 234 nurses and 54 doctors working in hospitals. A random cluster survey was carried out to obtain a sample of 146 health centers and nine hospitals.

In total, 185 doctors and 472 nurses working in public health establishments (hospitals and primary health centers) in the northern region of Morocco participated in the study.

Inclusion and exclusion criteria

Doctors and nurses working in primary healthcare establishments and those working in public hospitals were included in the study. Laboratory and radiology technicians, physiotherapists, and specialist doctors were excluded from the study as they do not directly interact with patients as frontline healthcare professionals. Also dietitians are not included, they could skew the data to obtain more correct answers on nutritional knowledge compared to other health personnel.

Study tools

The study used a self-administered questionnaire on sociodemographic characteristics and a multiple choice quiz on nutrition for diabetics, nutrition for high blood pressure and high cholesterol, nutrition for functional colopathy, nutrition for iron-deficiency anemia, nutrition for vitamin B12 deficiency, nutrition for overweight, nutrition for hyperuricemia or gout attack. The questionnaire was developed by a group of experienced dietitians based on similar studies inspired by the literature and standardized nutritional guides [13].The quiz was then evaluated by a group of experienced dietitians. The questionnaire was anonymous and took approximately 20 minutes to complete. It was prepared in English and French (Appendices 1, 2).

Study measures 

The scores of the answers to the questions corresponding to each of the diseases studied were calculated as well as the total score of all the answers. Response scores were described with median and interquartile range (IQR).

Ethical approval

The Tangier ethics committee (Faculty of Medecine and Pharmacy or Tangier-Morocco Ref. AC27AV/2022) gives exemption from opinion for observational and non-interventional studies. People who agreed to participate in the study signed their informed consent.

Statistical analyses

Statistical analyses were performed using SPSS statistical software version 21.0. Descriptive statistics were used to report the data. Scores are the average of correct responses where the true response receives 1 and the false response receives 0. Normality was examined by the Kolmogorov-Smirnov test with a p-value<0.05. The Mann Whitney test was used to test the association between median response scores and the main variables considered (gender, workplace, initial training, continuing training and receipt of nutritional information). Statistical tests were two-sided with a significance level of p<0.05. Spearmen’s correlation was used to identify the association between nutritional knowledge question response scores of participants (doctors and nurses) and their age. Multiple linear regression analysis was used to explore the most significant sociodemographic variables that may be associated with good nutritional knowledge.

## Results

Sociodemographic characteristics of respondents

This study includes 185 doctors and 472 nurses (Table 1). Concerning doctors, the sample is composed of 103 (55.7%) men and 82 (44.3%) women, with median age being 42 ± 5.32, and 181 (98%) answered the questions asked. 131 (70.8%) participants worked in primary healthcare centers and 54 (29.2%) in hospitals. 152 (82.2%) doctors said that they have not received any nutrition courses during their training, while 27 (14.6%) have followed continuing education in nutrition. Nearly a third of participants said they had received the Moroccan nutritional guides.

**Table 1:** Sociodemographic characteristics of participants *Receiving information: Reception of information on nutrition via official documents from the Ministry of Health, such as Moroccan guides to nutrition, national nutrition strategy guide, etc.

Variable	Doctors (N=185)	Nurses (N= 472)
Age (Year)		
26-36	16.2	44.1
37-47	68.6	40.9
48-55	15.1	15
Median Age	42±5.32	38.00±6.83
Sex (% of total)		
Female	44.3	56.4
Male	55.7	43.6
Work place (% of total)		
Primary healthcare center	70.8	50.4
Hospital	29.2	49.6
Courses on nutrition during training (% of total)		
Initial training	17.8	92.4
Continuing education	14.6	7.4
*Receiving information	29.7	31.6

For nurses, the sample includes 266 (56.4%) women and 206 (43.6%) men, with median age 38.00 ± 6.8, and 443 (94%) answered the questions asked. 238 (50.4)% responding nurses worked in primary healthcare centers and 234 (49.6%) in hospitals.

Most of the participating nurses (436 (92.4%)) testified that nutrition training was part of their basic training, while the majority of nurses (437 (92.6%)) stated that they had not followed continuing training in nutrition during their work. Only a third of them say they have received Moroccan nutritional guides. 

Assessment of nutritional knowledge of health professionals

Table 2 presents the scores and percentages of correct answer from doctors and nurses to questions on nutrition for diabetics, nutrition for high blood pressure and high cholesterol, nutrition for functional colopathy, nutrition for iron-deficiency anemia, nutrition for vitamin B12 deficiency, nutrition for overweight, nutrition for hyperuricemia or gout attack.

**Table 2:** Nutritional knowledge of doctors and nurses for diabetes, blood pressure and/or hypercholesterolemia, functional colopathy, iron-deficiency anemia, vitamin B12 (cobalamin) deficiency, overweight and gout attacks. * p<0.05; ** p<0.01; ***p<0.001

Variables	Score correct answers of nurses (Mean %)	Score correct answers of doctors (Mean %)	Score of total answers (Mean total %)	p-value
Knowledge about the nutrition for diabetes				
Which vegetables preferred	0.25 (25.5)	0.47 (47)	0.32 (36.25)	0.000 ***
Which vegetables moderate	0.16 (15.6)	0.31 (30.8)	0.20 (23.20)	0.000 ***
Which fruits preferred	0.16 (15.6)	0.40 (39.6)	0.22 (27.60)	0.000***
Which fruits limited	0.18 (18.4)	0.36 (35.7)	0.23 (27.05)	0.000***
Among bread, cereals, potatoes and legumes, which foods to control	0.27 (27.1)	0.71 (71.4)	0.40 (49.25)	0.000***
Which form of (bread, cereals, potatoes and legumes) preferred	0.09 (8.7)	0.39 (39)	0.17 (23.85)	0.000***
Which form of milk and dairy products preferred	0.17 (16.9)	0.22 (22.4)	0.18 (19.65)	0.107
Frequency of red meat consumption	0.53 (53.4)	0.78 (78)	0.60 (65.7)	0.000***
Frequency of chicken consumption (not fried)	0.46 (46.4)	0.55 (55.4)	0.49 (50.9)	0.038*
Frequency of fish consumption (not fried)	0.34 (34.3)	0.49 (49.2)	0.39 (41.75)	0.000***
Frequency of whole egg consumption	0.46 (45.6)	0.75 (75.1)	0.54 (60.35)	0.000***
Frequency of egg white consumption	0.35 (35.2)	0.46 (45.9)	0.38 (40.55)	0.011*
Dried fruit consumption	0.30 (29.9)	0.37 (37.3)	0.32 (33.60)	0.067
Light or diet products consumption	0.22 (21.6)	0.38 (37.8)	0.26 (29.70)	0.000***
Median of scores (% of total correct answers)	0.28 (28.15)	0.50 (47.5)	0.36 (37.82)	0.000***
Interquartile ranges	(0.07, 0.42)	(0.36, 0.64)	(0.14, 0.50)	
Knowledge about nutrition for high blood pressure and/or high cholesterol				
Which vegetables and fruits preferred	0.27 (27.5)	0.44 (44.3)	0.32 (35.90)	0.000***
Among bread, cereals, potatoes, and legumes, which foods preferred	0.22 (22)	0.55 (55.1)	0.31 (38.55)	0.000***
Which form of milk and dairy products recommended	0.22 (22)	0.56 (56.2)	0.32 (39.10)	0.000***
Among red meat and chicken, which foods are recommended	0.16 (16.5)	0.37 (37.3)	0.22 (26.90)	0.000***
Frequency of fish consumption	0.19 (18.7)	0.32 (31.7)	0.22 (25.20)	0.031*
Frequency of whole egg consumption	0.36 (36.4)	0.46 (45.6)	0.39 (41)	0.000***
Frequency of egg white consumption	0.26 (26.1)	0.45 (45.3)	0.31 (35.70)	0.000***
Salt and savory products consumption	0.48 (47.7)	0.86 (85.9)	0.58 (66.80)	0.000***
Median of scores (% of total correct answers)	0.28 (28.31)	0.57 (52.81)	0.28 (40.56)	0.000***
Interquartile ranges	(0, 0.43 )	(0.28, 0.71)	(0.14, 0.57)	
Knowledge about nutrition to relieve functional colopathy				
Which fruits, vegetables, and dried fruits preferred	0.09 (9.30)	0.26 (25.9)	0.14 (17.60)	0.000***
Which vegetables source of abdominal bloating	0.24 (24.30)	0.35 (34.6)	0.27 (29.45)	0.008**
Which vegetables to favor in case of predominance of diarrhea	0.08 (8.30)	0.27 (26.8)	0.13 (17.55)	0.000***
Among bread, cereals, potatoes, and legumes, which foods recommended in case of predominance of diarrhea	0.09 (8.70)	0.17 (16.7)	0.11 (12.70)	0.003**
Among bread, cereals, potatoes, and legumes, which foods recommended in case of predominance of constipation	0.06 (6.10)	0.16 (15.6)	0.9 (10.85)	0.000***
Which form of milk and dairy products preferred	0.04 (4.40)	0.25 (24.6)	0.10 (14.50)	0.000***
Recommended drinks	0.11 (11.20)	0.35 (35.1)	0.18 (23.15)	0.000***
Median of scores (% of total correct answers)	0 (10.32)	0,14 (25.6)	0 (17.97)	0.000***
Interquartile ranges	(0, 0.14 )	(0.14, 0.43 )	(0, 0.14)	
Knowledge about nutrition for iron-deficiency anemia				
Which fruits and vegetables to recommend for iron deficiency	0.23 (23.5)	0.30 (29.9)	0.25 (26.70)	0.092
Among meat, poultry, fish, and eggs, which food to recommend for iron deficiency	0.20 (19.7)	0.37 (36.7)	0.24 (28.20)	0.000***
Which legumes and dried fruits to recommend for iron deficiency	0.11 (10.6)	0.24 (24.3)	0.14 (17.45)	0.000***
Median of scores (% of total correct answers)	0 (17.93)	0.33 (30.3)	0 (24.11)	0.000***
Interquartile ranges	(0, 0.33)	(0, 0.67)	(0, 0.33)	
Knowledge about nutrition for vitamin B12 (cobalamin) deficiency				
Which food to recommend for vitamin B12 deficiency	0.7 (6.6)	0.29 (29.1)	0.13 (17.85)	0.000***
Median of scores (% of total correct answers	0 (6.6)	0 (29.1)	0.13 (17.85)	0.000
Interquartile ranges	(0, 0)	(0, 1)	(0, 0)	
Knowledge about nutrition for overweight				
Preferred form of vegetables and fruits	0.15 (15)	0.30 (29.7)	0.19 (22.35)	0.000***
Fruits portions should be control or not?	0.26 (26.1)	0.43 (42.7)	0.31 (34.40)	0.000***
Dried fruits consumption should be control or not?	0.23 (22.9)	0.31 (31.3)	0.25 (27.10)	0.026*
Among meat, poultry, fish, and eggs, which food to preferred	0.16 (16.3)	0.31 (31.5)	0.21 (23.90)	0.000***
Which form of milk and dairy products preferred	0.12 (12.5)	0.36 (36.2)	0.18 (24.35)	0.000***
Frequency of consumption of bread, cereals, potatoes, and legumes	0.09 (8.7)	0.22 (21.6)	0.12 (15.15)	0.000***
Among bread, cereals, potatoes, and legumes, which foods to moderate	0.18 (18.4)	0.37 (36.8)	0.24 (27.60)	0.000***
Preferred drinks	0.15 (15)	0.31 (30.8)	0.19 (22.90)	0.000***
Preferred cooking method	0.17 (17)	0.31 (30.9)	0.21 (24.05)	0.000***
Median of scores (% of total correct answers)	0.11 (16.9)	0.33 (32.38)	0.11 (24.64)	0.000***
Interquartile ranges	(0, 0.22)	(0.22, 0.44)	(0, 0.33)	
Knowledge about nutrition for gout attack				
All vegetables are allowed	0.09 (9.1)	0.19 (19.5)	0.12 (14.3)	0.000***
Which fruits preferred	0.08 (7.8)	0.18 (17.8)	0.11 (12.8)	0.000***
Among meat, poultry, fish, and eggs, which food to limit	0.11 (11.2)	0.24 (24.2)	0.15 (17.7)	0.000***
Among bread, cereals, potatoes, and legumes, which foods to preferred	0.14 (14.2)	0.29 (29.2)	0.18 (21.7)	0.000***
Recommended amount of dairy products should be reduced or increased?	0.08 (7.8)	0.18 (18.4)	0.11 (13.1)	0.000***
Which form of milk and dairy products preferred	0.07 (6.6)	0.16 (16)	0.09 (11.3)	0.000***
Drinks recommend	0.12 (11.7)	0.23 (22.8)	0.15 (17.25)	0.000***
Median of scores (% of total correct answers)	0 (9.77)	0.14 (21.12)	0.13 (15.45)	0.000***
Interquartile ranges	(0, 0.14)	(0.10, 0.28)	(0, 0.14)	
Median of total scores of answers to questions on nutritional knowledge in relation to secondary prevention (% of total correct answers)	0.14 (16.85)	0.32 (34.11)	0.17 (25.48)	0.000***
Interquartile ranges for median of total scores of answers to questions on nutritional knowledge in relation to secondary prevention	(0.07, 0.25)	(0.23, 0.39)	(0.09, 0.30)	

Concerning the nutritional knowledge of diabetics, the median score of correct response by doctors to the questions is 0.5 (IQR (0.36, 0.64)), for nurses is 0.28 (IQR (0.07; 0.42)). The total median score of correct answers from the both healthcare professionals is 0.36 (IQR (0.14, 0.50)).

Concerning nutrition for high blood pressure and/or hypercholesterolemia, the median score of correct answers from doctors is 0.57 (IQR (0.28, 0.71)), for nurses is 0.28 (IQR (0, 0.43)).The total median score of correct responses from both healthcare professionals is 0.29 (IQR (0.14, 0.57)).

Concerning nutrition for functional colopathy, the median of correct response score among doctors is 0.14 (IQR (0.14, 0.43)), for nurses is 0 (IQR (0, 0.14)). The median of the total scores of the correct answers of the both healthcare professionals is 0 (IQR (0, 0.14)).

Regarding diet for iron-deficiency anemia, the median of correct response score of doctors is 0.33 (IQR (0, 0.67)), for nurses is 0 (IQR (0, 0.33)). The median of the total scores of the correct answers of the both healthcare professionals is 0 (IQR (0, 0.33)).

Regarding diet for B12 deficiency, the median of doctors' correct answer scores is 0 (IQR (0, 1)), also for nurses is 0 (IQR (0, 0)). The median of the total scores of the correct answers of the both healthcare professionals is 0 (IQR (0, 0)).

Concerning nutrition for overweight, the median of correct answer score for doctors is 0.33 (IQR (0.22, 0.44)), for nurses is 0.11 (IQR (0, 0.22)). The median of the total scores of the correct answers of the both healthcare professionals is 0.11 (IQR (0, 0.33)).

Concerning nutrition of gout attacks or excess uric acid, the median of doctors' correct response scores is 0.14 (IQR (0.10, 0.28)), for nurses is 0 (IQR (0, 0.14)). The median of the total scores of the correct answers of the both health professionals is 0 (IQR (0, 0.14)).

**Figure 1:**
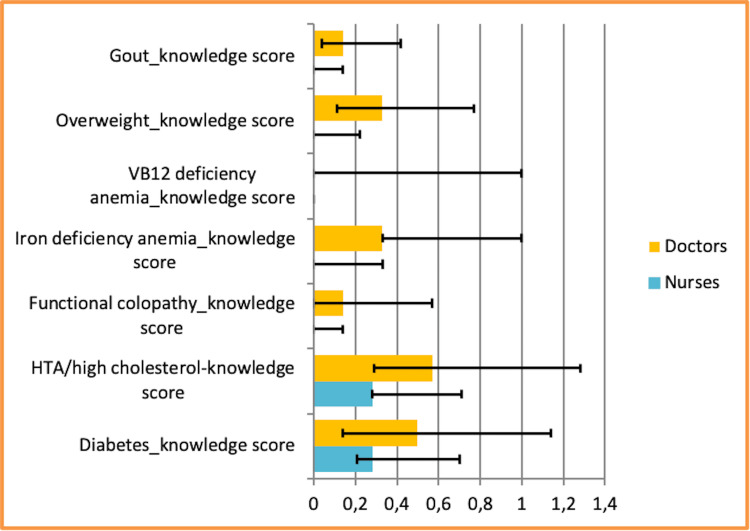
Distribution of nutritional knowledge scores of doctors and nurses on diabetes, blood pressure and/or hypercholesterolemia, functional colopathy, iron-deficiency anemia, vitamin B12 (cobalamin) deficiency, overweight, and gout attacks

The results of the study provide an overview of the level of knowledge of doctors and nurses in relation to secondary prevention. We can see from Figure 1 that the correct response scores among doctors are optimal compared to those of nurses.

Relationship between the median total score of health professionals' responses and their sociodemographic variables

In order to analyze the association between nutritional knowledge and sociodemographic Variables, the median of the total score of this knowledge in relation to secondary prevention of all respondents were calculated (Table 3).

**Table 3:** Association between the median of the total score of answers of health professionals (doctors and nurses) to questions on nutritional knowledge in relation to secondary prevention and their sociodemographic variables * p<0.05; ** p<0.01; ***p<0.001 *Receiving information: Reception of information on nutrition via official documents from the Ministry of Health, such as Moroccan guides to nutrition, national nutrition strategy guide, etc.

Variables	Correlation coefficient (r)	Median (Interquartile ranges)	Raw analysis p-value	Adjusted analysis p-value
Gender	-	0.17 (0.09, 0.30)	0.383	-
Age				
Doctors	0.244	42 (36.68, 47.32)	0.000	0.016*
Nurses		38 (31.17,44.83)		
Diploma				
Doctors	-	0.32 50.23 , 0.39)	0.000	0.000***
Nurses	-	0.14 (0.07 , 0.25)		
Workplace				
Primary healthcare center	-	0.24 (0.12 , 0.34 )	0.000	0.001**
Hospital	-	0.11 ( 0.04 , 0.22)		
Courses on nutrition during initial training				
Yes	-	0.14 ( 0.08 , 0.25)	0.000	0.000***
No	-	0.27 ( 0.18 , 0.36)		
Courses on nutrition during continuing education				
Yes	-	0.21 ( 0.14 , 0.47)	0.000	0.002**
No	-	0.16 ( 0.08 , 0.29)		
*Receiving information				
Yes	-	0.25 (0.10 , 0.35)	0.000	0.018*
No	-	0.15 (0.08 , 0.27)		

The present study determined a statistically significant association (p<0.001) between the median of the total scores of all participants' responses to nutritional knowledge in relation to secondary prevention and their diploma (doctors or nurses). In addition, the analysis reveals a statistically significant difference between health professionals who received a nutrition course during their basic and continuing training and those who did not (p<0.001 and p<0.001).

Additionally, median total nutrition knowledge scores were statistically significantly (p<0.001) associated with receipt of nutrition information.

Also, the workplace of doctors and nurses was statistically significantly associated with the median of total nutritional knowledge scores (p<0.001).

The results show that the age of the participants is positively correlated with the response score (r=0.244, p<0.001). No statistically significant differences in scores based on gender (p=0.383) were found. Multivariate analysis confirmed the results of bivariate tests regarding the association of correct answer score to nutrition questions in relation to secondary prevention and sociodemographic dimensions.

## Discussion

The objective of the current study was to assess the nutritional knowledge of doctors and nurses in the field of nutrition for hypertension and hypercholesterolemia, diabetes, overweight, functional colopathy, iron-deficiency anemia, vitamin B12 deficiency, and for gout attacks or excess uric acid.

The results obtained showed overall, whether doctors or nurses, a correct answer score to the questions concerning the seven areas covered were unsatisfactory. The median score for doctors' correct answers in all axes was significantly higher than that of nurses. But they are significantly associated with basic training, continuing education, information on nutrition, and their age.

657 participants (185 doctors and 472 nurses) were selected to assess their nutritional knowledge in relation to secondary prevention, using a multiple-choice questionnaire on nutrition for diabetes, high blood pressure and high cholesterol, digestive discomfort with functional colopathy, iron-deficiency anemia, vitamin B12 deficiency, overweight, and excess uric acid or gout attack.

The median total correct response score of all participants is 0.17 (IQR (0.9, 0.3)). The median of that of doctors is 0.32 (IQR (0.23, 0.39)) and that of nurses is 0.14 (IQR (0.07, 0.25)).

Among the seven nutritional content areas assessed in this study, we found that among doctors, the nutrition axis for high blood pressure and high cholesterol had the median correct response score of (0.57 IQR (0.28, 0.71)) followed by that of nutrition for diabetics (0.5 IQR (0.36, 0.64)), then the axis of nutrition for overweight (0.33 IQR (0.22, 0.44)) and iron-deficiency anemia (0.33 IQR (0, 0.67)), functional colopathy (0.14 IQR (0.14, 0.43 )), gout attack or excess uric acid (0.14 IQR (0.10, 0.28 )), and vitamin B12 deficiency (0 IQR ( 0,1)).

For nurses, the correct answer score of the nutrition axis for high blood pressure and hypercholesterolemia (0.28 IQR (0, 0.43)), and that of diabetes (0.28 IQR (0.07, 0.42)), are ranked first but with the lower scores than the doctors, followed by the nutrition axis for overweight (0.11 (0, 0.22)), and finally the nutrition axis for iron-deficiency anemia (0 IQR ((0, 0.33)), functional colopathy (0 IQR (0, 0.14)), gout attack or excess uric acid (0 IQR (0, 0.14)), and vitamin B12 deficiency (0 IQR (0, 0)).

The training curriculum for doctors is different from that for nurses, which could explain these differences in their knowledge. However, the latter, both among doctors and nurses, generally remain insufficient and must be improved.

In the present study, the median score of correct answers to questions on nutrition for diabetes among doctors was 0.5 IQR (0.36, 0.64), results lower than those found in Saudi Arabia in a cross-sectional study conducted among medical interns who graduated in 2019, 2020, and 2021 (0.56) [14]. In addition, in Qatar, the response score to questions on low glycemic index foods was 0.10, while in our study it was 0.47 [15]. Although, among nurses, the median of correct response score for diabetes nutrition questions was 0.28 (IQR (0.07, 0.42)), these were suboptimal results compared to those found in Ghana (0.44), and in South Korea (0.68) [16,17]. In India, the nurses indicated that a diabetic should not consume ripe fruits such as papaya and banana (0.58), which constitutes triple the score of correct answers in our study (0.18) [18]. 

As for the nutrition component for high blood pressure and hypercholesterolemia, doctors obtained a median of correct answer score of 0.57 (IQR (0.28, 0.71)) to the questions addressed in our research, optimal results compared to that found in the Saudi Arabia (0.44) [14]. In Iran, the correct answer score for questions related to foods to reduce for hypertension and hypercholesterolemia, such as egg yolk, offal, fatty meats, and full-fat dairy products, was 0.50. Additionally, the response score for questions about replacing high-fat, red, and processed meats with legumes, fish, and poultry, as well as reducing salt consumption, is 0.25 [19]. However, in our study, the correct response score of the questions on recommended dairy products was 0.56, the fatty meats, offal and fish, poultry and eggs was 0.37, and the cereal products and legumes was 0.55. In addition, the response score on the low-sodium diet was recommended for a hypertensive person (0.86). As for nurses, the median score for correct answers to questions on nutrition in cases of high blood pressure and high cholesterol was 0.28 (IQR (0, 0.43)), this result was almost half of that found in Jordan (0.55), where the score of nurses who knew that fish consumption is recommended to reduce hyperlipidemia was 0.78, while the score of our nurses who identified the frequency of fish consumption in the context of high blood pressure and hypercholesterolemia was only 0.19 [20]. 

Concerning nutrition for obesity, the median of nurses’ correct answer score was 0.28 (IQR (0.07, 0.42)), which represents one-third of the answer score found in South Korea (0.54) [17]. In Jordan, the correct answer score was (0.59); however, the response score to the question regarding the recommendation of dietary fiber in cases of obesity was 0.91 [20]. This contrasts with our results, where the response score for the frequency of consumption of cereals and legumes was 0.09, and the score for the consumption of fruits and vegetables was 0.15. Additionally, the majority of nurses believe there is a need to reduce carbohydrates and animal fats, with scores of 0.88 and 0.74, respectively, to lose weight [20]. This result is close to our correct answer score, for the question on meats (0.16) and recommended dairy products (0.12). As for doctors, the median of correct answer scores to questions on nutrition for obesity was 0.5 (IQR (0.36, 0.64)), a result lower than that observed in Saudi Arabia (0.54) [14]. In Iran, the response score to questions on moderation of vegetables and legumes was 0.72, consumption of whole grains was 0.82, restriction of high-fat dairy products was 0.97 [19]. These results are very optimal compared to that concluded by our research, the highest score was obtained by the question on the portions of fruit to control 0.43, then 0.31 obtained by the question on the drinks recommended for obese people.

As for the nutritional component of functional colopathy, the median of doctors' good knowledge scores was 0.14 (IQR (0.14, 0.43)), a suboptimal result compared to that reported in in Ireland (0.78) [21]. While for nurses, the median score for correct answers to questions on nutrition for functional colopathy was 0 (IQR(0, 0.14)), results lower than that were found in Ireland (0.52 ). 

Regarding recommended nutrition in vitamin B12 deficiency, the median correct answer score among doctors was 0 (IQR (0.1)). Result lower than that was found in Croatia (0.14) [22]. However, the results in Saudi Arabia (0.31) were very optimal in comparison with our findings [23]. For nurses, the median score of correct knowledge on food sources of vitamin B12 is 0 (IQR (0, 0)), this result is very low compared to that recorded in Australia (0.12) [24]. However, in a study conducted in New Zealand, the correct answer score that seafood, eggs, and dairy products are rich in vitamin B12 was 0.28 [25]. 

Concerning nutrition for iron-deficiency anemia, the median of doctors' correct response scores to the questions addressed in our study was 0.33 (IQR (0, 0.67)), this figure is suboptimal to that found in Ethiopia (0.59) [26]. Regarding nurses, the median score for correct answers to questions about foods recommended for iron-deficiency anemia is 0 (IQR (0, 0.33)). This score is suboptimal compared to that found in Australia (0.48) [24]. In this country, the response score for iron-rich legumes is 0.59, whereas in our study, it is only 0.11.

For nutrition in cases of excess uric acid or gout attacks, the median score of doctors' correct answers to the questions asked is 0.14 (IQR (0.10, 0.28)), this score lower than that reported in Saudi Arabia (0.63) [27]. Concerning nurses, the median score for correct answers to questions on the nutrition of people suffering from gout or hyperuricemia was 0 (IQR (0, 0.14)), this result was very far from that found in India (0.53) [18].

Our results do not identify a statistically significant association of the total score of correct answers in terms of nutritional knowledge in terms of secondary prevention of all participants (doctors and nurses), with their sex (p=0.383), but it is significant with their age (r=0.244, p=0.016). This result corroborates similar research, this can mean that professionals gain knowledge through experience and practice [28] .

Our research indicates that only 17.8% of doctors benefited from nutrition courses during their basic training and 14.6% during their continuing training. These results are consistent with those of research carried out in the United States, which indicated that only 25% of medical schools integrate certain nutrition elements into their courses [29].

In our study, 92% of nurses reported taking a 40-hour nutrition course during their basic training. However, the response score indicated insufficient preparation to provide nutrition education to the population. This result coincides with the literature that reported that out of the 90% of respondents who received a nutrition course during their basic training, 76% were dissatisfied with the time devoted to nutrition education and 40% were insufficiently prepared to provide nutritional care [30].

Also, the present study shows that the total score of responses to questions on nutrition in relation to secondary prevention is significantly associated with basic training among the two health professionals (p<0.001). These results corroborate those of the literature [28]. In addition to a statistically significant relationship of this score with continuing education (p=0.002), this result is similar to those of a multitude of studies [24].

Regarding receipt of nutritional information from ministerial and other official sources, only 32% of participants in our study reported having received ministerial guides on nutrition, while 68% of health professionals lacked adequate resources to access the information in the field of nutrition. In contrast, in New Zealand, researchers reported that 47% of midwives used Ministry of Health materials and 53% contacted dietitians for information [25]. 

The present study identified a significant association (p=0.018) between receipt of nutritional information and the median total score of all participants' responses to nutritional knowledge in relation to secondary prevention. This finding parallels the finding that receiving information about new nutritional guidelines was statistically significantly associated with good nutritional knowledge [16].

Our study found that there was a significant association between workplace and total response score of doctors and nurses (p=0.001), a result similar to those of the literature [28]. This could be explained by the high rate of access (78.8%) of health-center professionals to information, in particular guides and documents from the Ministry of Health. This finding is similar to that of a study which found a significant difference between the good nutritional knowledge of midwives working in regional hospitals and those working in rural hospitals [24]. 

The limitation of this study is that private-sector professionals were not included in our study. Furthermore, the study was conducted in the northern region of Morocco. Although basic training is unified, continuing training depends on the continuing training unit of the regions. 

Young doctors and nurses with less experience were relatively overrepresented in the sample. This could be attributed to selection bias, as it was difficult to obtain consent from older doctors and nurses. As a result, our study was limited to 26 public sector professionals.

## Conclusions

In conclusion, this study highlighted the major gaps in the nutritional knowledge of doctors and informants in relation to the secondary prevention of NCDs. However, health professionals working in primary healthcare establishments have good nutritional knowledge in relation to secondary prevention of NCDs than those working in hospitals but less than expected. Since primary care physicians and nurses are the first line of defense in healthcare, they should undergo continuing nutrition education programs to ensure safe and sound nutritional advice not only to patients but also to the audience.

Our results showed that the nutritional knowledge of doctors and nurses in Morocco still needs to be improved, and that policy makers should devote systematic efforts to basic and continuing nutritional education in medical and nursing faculties, which will provide doctors and nurses with an adequate level of nutritional knowledge and able to advise the population.

## Appendices

Appendix 1 

**Table 4:** Assessment of nutritional knowledge of health professionals in the TTA region TTA: Tangier-Tetouan-AlHoceima

Sociodemographic characteristics			
	Your gender: Female □ Male □		
	Your age (years) : ……. |__|__| |__|__|__|__|		
	Your diploma Doctorate in general medicine □ License in Nursing/Midwifery □		
	Your function Doctor in a hospital □ Doctor in a health center □ Doctor in a private practice □ Nurse/Midwife in a center health □ Nurse/Midwife in a hospital □		
	Initial training Did you study a nutrition module during your basic training? Yes □ No □		
	Continuing education During your continuing education, did you follow nutrition training? Yes □ No □		
	Reception of nutritional information Do you receive information on nutrition: Official documents (Moroccan nutrition guide, nutrition strategy, etc.)? Yes □ No □		
Evaluation of nutritional knowledge in relation to pathological cases			
	Nutritional knowledge for people with diabetes		
	What are the nutritional recommendations for a diabetic		
	Fruits and vegetables	The preferred vegetables are Raw vegetables □ Cooked vegetables □ I don't know □ Which of the following vegetables should be moderated? Cooked carrots □ Cooked beets □ Pumpkin □ Cooked turnips □ Cooked beans □ Peas □ Zucchini □ Tomato □ Broccoli □ I don’t know □ Favor the consumption of the following fruits: Apple □ Pear □ Cherries □ Pomegranate □ Peach □ Orange □ Pineapple □ Strawberries □ Raspberries □ I don't know □ Which of the following fruits should be limited? Dried figs □ Banana □ Grapes □ Papaya □ Mango□ Melon □ Pineapple □ Watermelon □ Cherries □ Apple □ Strawberries □ Grapefruit □ I don’t know □	
	Breads, potato, cereals and dried vegetables	Foods whose quantity consumed must be controlled: Bread □ Cereals □ Potato □ Dried vegetables □ I don't know □ Favor the following foods: Fresh bread □ Toasted bread □ White bread □ Whole meal bread □ Barley bread □ Overcooked pasta □ Undercooked pasta (al dente) □ I don’t know □	
	Milks and dairy products (yogurts, cheeses)	Choose the following product: Semi-skimmed milk □ Whole milk □ Raib □ Leben □ Fresh cheese □ Hard cheese □ I don’t know □	
	Meat and poultry, fish, eggs	The frequency of consumption of animal proteins is: Maximum lean red meat: 1 time / day □ 2 times / week □ I don't know □ Unfried chicken 1 time / day □ 2 times / week □ I don't know □ Non-fried fish: 1 time / day □ 3 times / week □ I don't know □ Maximum whole eggs: 1 time / day □ 2 times / week □ I don't know □ Egg white can be consumed: Every day □ 2 times/week □ I don't know □	
	Dried fruits	Consumption of dried fruits should be: Privileged □ Limited □ I don't know □	
	Light or diet products	Light or diet products should be? Privileged □ Limited □ I don't know □	
	Nutritional knowledge for high blood pressure and/or high cholesterol		
	What are the nutritional recommendations in case of hypertension and/or hypercholesterolemia?		
	Fruits and vegetables	The foods to favor are: Raw vegetables □ Cooked Vegetables □ Raw Fruits □ Canned Vegetables and Fruits □ I don’t know □	
	Whole grains, potatoes and pulses	Prioritize the consumption of Wheat bran □ Whole grain cereals □ Refined cereals □ Pulses □ I don’t know □	
	Milk and dairy products (yogurts, cheeses)	Recommend the consumption of : Plain yogurt □ Plain white cheese □ 2% semi-skimmed milk □ Hard cheese (Edam) □ Whole milk □ I don’t know □	
	Meat and poultry, fish, eggs	Advise the consumption of Lean red meat □ Turkey □ Chicken without skin □ Sheep meat □ White fish □ Oily fish □ Offal □ I don't know □ Advise the consumption of White fish □ Oily fish □ I don't know □ Whole eggs consumed as much as possible: 1 time / day □ 2 times / week □ I don't know □ Egg white can be consumed: Every day □ 2 times/week □ I don't know □	
	Salt and savory products	In case of hypertension, you must limit the consumption of the following foods: Hard cheese (Edam) □ Olive □ Khlii □ Sausages □ Charcuterie □ Plain white cheese □ I don’t know □	
	Nutritional knowledge for people with digestive discomfort and functional colopathy?		
	What foods do you recommend to relieve functional colopathy?		
	Fruits and vegetables and dried fruits	In case of intestinal discomfort, you should prioritize: Raw vegetables □ Cooked vegetables □ Raw fruits □ Dried fruits □ Cooked or ripe fruits □ Red fruits □ Citrus fruits □ I don’t know □ Vegetables that are a source of abdominal bloating are: Cabbage □ Turnips □ Onions □ Artichokes □ Beetroot □ Mushrooms □ Peas □ Carrot □ Zucchini □ Cucumber □ I don't know □ Vegetables to favor in case of predominance of diarrhea are: Carrot □ Zucchini □ Beetroot □ Eggplant □ Green beans □ Peas □ Artichokes □ I don’t know □	
	Breads, potato, cereals and dried vegetables	Foods recommended in cases of predominantly diarrhea are: Oats □ Barley □ Whole wheat □ Potatoes□ Wheat bran □ I don’t know □ The foods recommended in cases of predominant constipation are: Lentils □ Red beans □ White beans □ Chickpeas □ Whole meal bread □ Wheat bran □ Rice □ Potatoes□ I don't know □	
	Milks and dairy products (yogurts, cheeses)	The preferred dairy products are : Skimmed milk □ Whole milk □ Fermented milk □ Plain yogurt □ Cottage cheese □ Hard cheese □ Soft cheese □ I don’t know □	
	Drinks	Recommended drinks are: Tap water □ Mineral water □ Soda □ Coffee with milk □ Fruit juice □ Tea □ Herbal tea □ Coffee □ I don’t know □	
	Nutritional knowledge for people anemic due to iron deficiency		
	What foods are recommended for a person with iron anemia?		
	Fruits and vegetables	Raw spinach □ Purslane ( Rejla ) □ Cabbage □ Peas □ Parsley □ Pepper □ Beans □ Beetroot □ I don’t know □	
	Meat and poultry, fish, eggs	Red meat □ Beef liver □ Poultry liver □ Heart □ Spleen □ Shrimp □ Sardine □ Anchovies □ Egg yolk □ I don’t know □	
	Legumes, dried fruits	Legumes □ Dried fruits □ Oleaginous fruits (avocado, olive, almond, etc.) □ I don’t know □	
	Nutritional knowledge for people anemic due to vitamin B12 (cobalamin) deficiency		
	What foods are recommended for vitamin B12 anemia?		
	Foods to favor in case of B12 deficiency anemia: Red meat □ Poultry □ Eggs □ Offal □ Fish □ Dairy products □ Vegetables and fruits □ Cereals □ Pulses □ I don't know □		
	Nutritional knowledge for overweight people		
	What foods should we recommend to overweight people?		
	Vegetables, fruits and dried fruits	Vegetables and fruits to consume in the form of: Raw □ Cooked □ Frozen □ Canned □ I don’t know □ Control the portions of fruit consumed Yes □ No □ I don’t know □ Consumption of: oilseed fruits: walnuts, hazelnuts, almonds is A Privileged □ To be Limited □ I don’t know □	
	Meats, poultry, fish, eggs	The foods to favor in case of overweight are: Rabbit □ Lean roast meat □ Heart □ Liver □ Grilled poultry without skin □ Fried poultry □ Mutton □ Cold meats □ Kidneys □ Fried fish □ Hamburgers □ Snacks □ Dishes with sauces □ I don’t know □	
	Milks and dairy products (yogurts, cheeses)	The preferred dairy products are: Skimmed milk □ Natural white cheese □ Natural yogurt □ Soft cheeses (Camembert) □ Whole milk □ Hard cheese (Edam) □ Processed cheese (the laughing cow) □ I don’t know □	
	Bread, potato cereals and dried vegetables	The frequency of consumption of: bread, cereals, potatoes and pulses is: 1 time/day □ Every meal □ I don’t know□ Foods to moderate if you are overweight: Wholemeal bread and cereals□ Potato □ Sweet potato □ Pulses □ I don’t know □	
	Drinks	Recommended drinks are: Tap water □ Coffee □ Black tea □ Green tea □ Pure fruit juice □ Sweetened drink □ I don’t know □	
	Cooking method	The preferred cooking method in case of excess weight is: Baking at low temperature □ Cooking in a pressure cooker (high temperature and high pressure) □ Minimal cooking (half-cooked vegetables) □ Overcooking vegetables (well-cooked vegetables) □ · Steam cooking □ · Cooking pasta al dente (not overcooked) □ · Tagine cooking (slow with low heat) □ · Frying cooking □	
	Nutritional knowledge in case of excess uric acid and “gout” attacks		
	Fruits and vegetables	All vegetables are allowed: Yes □ No □ I don't know □ The fruits to favor are: Fresh fruit □ Frozen fruit □ Pressed fruit □ All dried fruits (dates, grapes, prunes.) □ Dried nuts (walnuts, hazelnuts) □ I don't know □	
	Meats, poultry, fish, eggs	Foods whose consumption should be limited: Poultry □ Offal □ Eggs □ Seafood □ Blue fish □ White fish □ Mutton □ I don't know □	
	Bread, potato and legume cereals	The foods to favor are: Whole grains □ Refined grains □ Potatoes □ Legumes □ I don’t know □	
	Milks and dairy products (yogurts, cheeses)	The recommended amount of dairy products should be: Increased □ Decreased □ I don’t know □ The preferred products are: Whole milk □ Soft cheeses □ Hard cheese □ Skimmed milk □ Low-calorie yogurt □ I don't know □	
	Beverage	Recommended drinks are: Tap water □ Coffee □ Black tea □ Green tea □ Fruit juice □ Sweet drink □ I don’t know □	

Appendix 2

**Table 5:** Évaluation des connaissances nutritionnelles des professionnels de santé de la région TTA TTA: Tangier-Tetouan-AlHoceima

I. Caractéristiques sociodémographiques		
1.	Votre sexe : Féminin □ Masculin□	
2.	Votre âge (ans) : ……. |__|__| |__|__|__|__|	
3.	Votre diplôme Doctorat en médecine générale□ Licence en soins infirmiers/Sage-femme □	
4.	Votre fonction Médecin dans un hôpital □ Médecin dans un centre de santé □ Médecin dans un cabinet privé □ Infirmier /Sage -femme dans un centre de santé □ Infirmier /Sage -femme dans un hôpital □	
5.	Formation initiale Avez-vous étudié un module de nutrition durant votre formation de base ? Oui □ Non □	
6.	Formation continue Lors de votre formation continue avez-vous suivi une formation en nutrition ? Oui □ Non □	
7.	Réception d’information nutritionnelle Recevez-vous l’information en matière de nutrition : Documents officiels (guide marocain de nutrition, la stratégie de nutrition…) ? Oui□ Non□	
II. Évaluation des connaissances nutritionnelles en relation avec des cas pathologiques		
A.	Connaissances nutritionnelles pour les personnes diabétiques	
	Quelles sont les recommandations nutritionnelles pour un diabétique	
	Fruits et légumes	Les légumes à privilégier sont Légumes crus□ Légumes cuits□ Je ne sais pas□ Lesquels des légumes suivants à modérer ? Carottes cuites □ Betteraves cuites □ Potiron□ Navets cuits□ Fèves cuites□ Petits pois□ Courgette □ Tomate □ Brocoli □ Je ne sais pas□

		Privilégier la consommation des fruits suivants : Pomme□ Poire□ Cerises □ Grenade□ Pêche □ Orange□ Ananas□ Fraises□ Framboises□ Je ne sais pas□ Lesquels des fruits suivants à limiter ? Figues séchées□ Banane□ Raisin□ Papaye□ Mangue□ Melon□ Ananas □ Pastèque□ Cerises □ Pomme□ Fraises□ Pamplemousse□ Je ne sais pas□
	Pains, céréales pomme de terre et légumes secs	Les aliments dont la quantité consommée doit être contrôlée : Pain □ céréales□ pomme de terre □ légumes secs□ Je ne sais pas□ Privilégier les aliments suivants : Pain frais□ Pain grillé□ Pain blanc□ Pain complet□ Pain à l’orge□ Pâtes trop cuites□Pâtes pas trop cuites(al dente)□ Je ne sais pas□
	Laits et produits laitiers (yaourts, fromages)	Privilégier le produit suivant : Lait demi écrémé□ Lait entier□ Raib □ Leben□ Fromage frais □ Fromage à pâte dure□
	Viandes et volailles, poissons,œufs	La fréquence de consommation des protéines animales est de : Viande rouge maigre maximum : 1 Fois / jour□ 2 Fois /semaine□ Je ne sais pas□ Poulet non frite 1 Fois / jour□ 2 Fois /semaine□ Je ne sais pas□ Poissons non frites : 1 Fois / jour□ 3 Fois /semaine□ Je ne sais pas□ Œufs entiers maximum : 1 Fois / jour□ 2 Fois /semaine□ Je ne sais pas□ Le blanc d’œuf peut être consommé : Chaque jour□ 2 Fois /semaine□ Je ne sais pas□
	Les fruits secs	Consommation des fruits secs doit être : Privilégiée□ Limitée □ Je ne sais pas□
	Produits lights ou de régime	Produits lights ou de régime doivent être ? Privilégié□ Limité □ Je ne sais pas□
B.	Connaissances nutritionnelles pour l’ hypertension artérielle et/ou l’hypercholestérolémie	
	Quelles sont les recommandations nutritionnelles en cas de HTA et/ou l’hypercholestérolémie?	
	Fruits et légumes	Les aliments à privilégier sont : Légumes crus □ Légumes cuits□ Fruits crus□ Légumes et Fruits en conserve □ Je ne sais pas □

	Céréales complets, pomme de terre et légume secs	Privilégier la consommation de Son de blé□ Céréales à grains entiers□ Céréales raffinées □ Légume secs□ Je ne sais pas□
	Lait et produits laitiers (yaourts,fromages)	Recommander la consommation de : Yaourt nature□ Fromage blanc nature□ Lait demi écrémé à 2 % □ Fromage dure(Edam)□ Lait entier□ Je ne sais pas□
	Viandes et volailles, poissons,œufs	Conseiller la consommation de Viande rouge maigre□ Dinde□ Poulet sans la peau□ Viande du mouton □ Poisson blancs□ Poissons gras □ Abats □ Je ne sais pas□ Conseiller la consommation de Poisson blancs□ Poissons gras □ Je ne sais pas□ Œufs entiers consommé au maximum : 1 Fois / jour□ 2 Fois /semaine□ Je ne sais pas□ Le blanc d’œuf peut être consommé : Chaque jour□ 2 Fois /semaine□ Je ne sais pas□
	Sel et produitssalés	Pour une personne avec HTA il lui faut un régime hyposodé : En permanence□ Occasionnelle □ Je ne sais pas□ Encas d’HTA, il faut limiter la consommation des aliments suivants : Fromage dure (Edam)□ Olive □ Khlii□ Saucisses□ Charcuterie □ Fromage blanc nature□ Je ne sais pas □
C.	Connaissances nutritionnelles pour les personnes inconfort digestif avec colopathie fonctionnelle ?	
	Quels sont les aliments que vous conseillez pour soulager la colopathie fonctionnelle ?	
	Fruits et légumes et fruits secs	En cas d’inconfort intestinal, il faut privilégier : Légumes crus □ Légumes cuites□ Fruits crus □ Fruits secs□ Fruits cuites ou mûrs □ Fruits rouges□ Agrumes □ Je ne sais pas□ Légumes source de ballonnement abdominale sont : Choux□ Navets□ Oignons□ Artichauts□ Betterave□ Champignons□ Petits pois □ Carotte□ Courgette□ Concombre□ Je ne sais pas□ Légumes à privilégier en cas de prédominance de diarrhée sont : Carotte□ Courgette□ Betterave□ Aubergine□ Haricots verts□ Petits pois□ Artichauts□ Je ne sais pas□

	Pains, céréales pomme de terre et légumes secs	Les aliments conseillés en cas de prédominance de diarrhée sont : Avoine□ Orge□ Blé entier□ Pommes de terre□ Son de blé□ Je ne sais pas□ Les aliments conseillés en cas de prédominance de constipation sont : Lentilles□ Haricots rouges□ Haricots blancs□ Pois chiches□ Pain complets □ Son de blé □ Riz □ Pommes de terre□ Je ne sais pas□
	Laits et produits laitiers (yaourts, fromages)	Les produits laitiers à privilégier sont : Lait écrémé□ Lait entiers □ Lait fermenté□ Yaourt nature□ Fromage blanc □ Fromage à pâte dur □ Fromage à pâte molle □ Je ne sais pas□
	Boissons	Les boissons à conseiller sont : Eau de robinet□ Eau minéral□ Soda□ Café au lait□ Jus de fruits □ Thé □ Tisane□ Café□ Je ne sais pas□
D.	Connaissances nutritionnelles pour les personnes anémiques par carence en Fer	
	Quels sont les aliments recommandés pour une personne avec l’anémie en fer ?	
	Fruits et légume	Épinard cru□ Pourpier (Rejla) □ Choux□ Petits pois□ Persil□ Poivron□ Fèves□ Betterave□ Je ne sais pas□
	Viandes et volailles, poissons,œufs	Viande rouge□ Foie du bœuf □ Foie du volaille□ Cœur □ Rate□ Crevette □ Sardine□ Anchois□ Jaune d’œuf □ Je ne sais pas□
	Légumineuse, fruits secs	Légumineuse □ Fruits secs□ Fruits oléagineux (avocat, olive, amande…) □ Je ne sais pas□
E.	Connaissances nutritionnelles pour les personnes anémiques par carence en vitamine B12 (cobalamine)	
	Quels sont les aliments recommandés en cas d’anémie en vitamine B12 ?	
	Les aliments à privilégier en cas d’anémie par déficit en B12 : Viande rouge □ Volaille □ Œufs □ Abats □ Poissons □ Produits laitiers□ Légumes et fruits□ Céréales□ Légumes secs□ Je ne sais pas□	
F.	Connaissances nutritionnelles pour les personnes en surpoids	
	Quels aliments doit-on conseiller aux personnes en surpoids ?	
	Légumes, fruits et fruits secs	Les légumes et des fruits à consommer sous forme : Crus□ Cuits □ Surgelés□ En conserve□ Je ne sais pas□ Contrôler les portions de fruits consommées Oui □ Non □ Je ne sais pas □ La consommation des : fruits oléagineux : noix, noisettes, amandes est A Privilégiés □ A Limiter □ je ne sais pas□
	Viandes, volailles, poissons, œufs	Les aliments à privilégier en cas de surpoids est :

		Lapin□ Rôti de viande maigre□ Cœur□ Foie□ Volaille grillée sans la peau □ Volaille frite □ Viande du mouton □ Charcuterie□ Rognons (reins) □ Poissons frite □ Hamburgers□ Snacks□ Plats en sauces□ Je ne sais pas□
	Laits et produits laitiers (yaourts, fromages)	Les produits laitiers à privilégier sont : Lait écrémé□ Fromage blanc naturel □ Yaourt nature□ Fromages à pâte molle (Camembert) □ Lait entier □ Fromage fondu (la Vache qui rit) □ Fromage dur(Edam)□ Je ne sais pas□
	Pain, céréales pomme de terre et légumes secs	La fréquence de consommation de : pain, céréales, pomme de terre et légumes secs est de : 1 Fois /jour □ A Chaque repas □ Je ne sais pas □ Les aliments à modérer en cas de surpoids : Pain complet et les céréales □ Pomme de terre□ Patate douce □ Légumes secs□ Je ne sais pas □
	Boissons	Les boissons recommandées sont : Eaux de robinet □ Café□ thé noir□ thé vert □ Jus de fruits pur □ Boisson sucré□ Je ne sais pas□
	Mode de cuisson	Le mode de cuisson à privilégier en cas de surpoids est : Cuisson au four à basse température□ Cuisson en cocotte-minute (température élevée et forte pression) □ Cuisson à minima (légumes mi- cuits) □ Cuisson excessive des légumes (légumes bien cuits) □ Cuisson à la vapeur□ Cuisson al dente des pates (pas trop cuites) □ Cuisson en tagine (lente avec feu douce) □ Cuisson en friture □
G	Connaissances nutritionnelles en cas d´acide urique en excès et crise de « goutte »	
	Fruits et légumes	Tous les légumes sont autorisés : Oui□ Non□ Je ne sais pas□ Les fruits à privilégier sont : Fruits frais□ Fruits surgelés□ Fruits pressé□ Tous les fruits secs (dattes, raisins, pruneaux.) □ Fruits secs à coque (noix, noisettes) □ Je ne sais pas□

	Viandes, volailles, poissons, œufs	Les aliments dont la consommation doit être limitée : Volailles□ Abats□ Œufs□ Fruits de mer□ Poissons bleus□ Poissons blancs□ Viande du mouton□ Je ne sais pas□
	Pain, céréales pomme de terre et légumineuse	Les aliments à privilégier sont : Céréales complet□ Céréales raffinées□ Pommes de terre□ Légumineuse□ Je ne sais pas□
	Laits et produits laitiers (yaourts, fromages)	La quantité recommandée en produits laitiers doit être : Augmentée □ Diminuée□ Je ne sais pas□ Les produits à privilégier sont : Lait entier□ Fromages à pâte molle□ Fromage dur□ Lait écrémé □ Yaourt hypocalorique □ Je ne sais pas□
	Boisson	Les boissons recommandées sont : Eaux de robinet □ Café□ Thé noir□ Thé vert □ Jus de fruits□ Boisson sucré□ Je ne sais pas□
